# Effect of Rhamnolipids on Microbial Biomass Content and Biochemical Parameters in Soil Contaminated with Coal Tar Creosote

**DOI:** 10.1515/biol-2019-0060

**Published:** 2019-12-31

**Authors:** Arkadiusz Telesiński, Ariel Brito Zambrana, Grzegorz Jarnuszewski, Kornel Curyło, Teresa Krzyśko-Łupicka, Barbara Pawłowska, Krystyna Cybulska, Jacek Wróbel, Marek Rynkiewicz

**Affiliations:** 1 Department of Plant Physiology and Biochemistry, West Pomeranian University of Technology, Szczecin, 17 Słowackiego St. 17, 71-434 Szczecin, Poland; 2 Faculty of Science and Technology, Universidad Autónoma Gabriel Rene Moreno, FACET Avenida Busch entre 2do/3er anillo, Santa Cruz, Bolivia; 3 Department of Soil Science, Grassland and Environmental Chemistry, West Pomeranian University of Technology, Szczecin, 17 Słowackiego St. 17, 71-434 Szczecin, Poland; 4 Independent Department of Biotechnology and Molecular Biology, University of Opole, 6a Kardynała Kominka St., 45-035 Opole, Poland; 5 Department of Biochemistry, Biotechnology and Ecotoxicology, Jan Długosz University in Częstochowa, 13/15 Armii Krajowej Av., 42-200 Częstochowa, Poland; 6 Department of Chemistry, Microbiology and Environmental Biotechnology, West Pomeranian University of Technology in Szczecin, 17 Słowackiego St., 71-434 Szczecin, Poland; 7 Department of Construction and Use of Technical Device, West Pomeranian University of Technology, 3 Papieża Pawła VI St., 71-459 Szczecin, Poland

**Keywords:** bioremediation, polycyclic aromatic hydrocarbons, sandy clay loam, soil enzymatic activity

## Abstract

The objective of the present study was to compare the effect of rhamnolipids on the microbial biomass content and the activity of dehydrogenases (DHA), acid phosphatase (ACP), alkaline phosphatase (ALP), and urease (URE) in soil contaminated with two types of coal tar creosote: type C and type GX-Plus. The experiment was carried out on samples of sandy clay loam under laboratory conditions. Coal tar creosote was added to soil samples at a dose of 0 and 10 g·kg^−1^ DM, along with rhamnolipids at a dose of 0, 10, 100, and 1000 mg·kg^−1^ DM. The humidity of the samples was brought to 60% maximum water holding capacity, and the samples were incubated at 20°C. Microbial and biochemical parameters were determined on days 1, 7, 21, and 63. The obtained results demonstrated that the addition of rhamnolipids did not result in any significant changes in the activity of the determined parameters in the uncontaminated soil. However, it was observed that the application of these biosurfactants, particularly at the dose of 1000 mg·kg^−1^ DM, largely decreased the effect of coal tar creosote on the determined parameters. Moreover, the microbial biomass and the activity of ALP and URE were found to be the best indicator of bioremediation of soil contaminated with coal tar creosote.

## 1 Introduction

Coal tar creosote is a mixture of coal tar distillation products, and its boiling temperature ranges from 200°C to 360°C [1]. It consists of various aromatic compounds. The largest group of coal tar creosote compounds includes components with neutral characteristics (80%–90%). These components are primarily polycyclic aromatic hydrocarbons (PAHs), e.g., naphthalene, anthracene, phenanthrene, pyrene, chrysene, etc. [2]. The content of acidic components, primarily phenols, typically ranges from 4% to 16%. The basic components typically constitute 3.5%–4.5% and normally include pyridine and its derivatives, quinoline and its methyl derivatives, isoquinoline, and so forth [3].

For many years, coal tar creosote was used to impregnate wooden railroad ties and telephone poles [4]. Following the regulation (no. 528/2012) of the European

Union and European Council dated May 22, 2012, coal tar creosote has been identified as a non-threshold carcinogen classified as a 1B category carcinogenic agent [5]. At present, only coal tar creosote with the benzo(a) pyrene (BaP) content of less than 50 mg·kg^−1^ is allowed for use. According to the Western European Institute for Wood Preservation (WEI-IEO), this amount of BaP is also a characteristic of type C coal tar creosote, which is obtained from the coal tar fraction with medium boiling point and low odor intensity. GX-Plus type is a mixture of coal tar creosote type C and mineral oils [6].

Several PAHs exhibit carcinogenic properties; moreover, they have been shown to induce numerous biological mutations [7]. The remaining compounds classified in this group, despite not showing carcinogenic properties, may intensify the synergistic effect [8]. Hence, the presence of these compounds in the environment has become the topic of interest of many researchers because of constant exposure of humans to them.

Soil is one of the most important elements of the environment, which accumulates considerable amounts of hydrophobic organic contaminants such as PAHs. After penetrating into the soil, these compounds may be taken up by plants and they may also penetrate into surface and ground waters. PAHs in soil exhibit poor mobility and high durability, and prolonged soil contamination with these compounds results in their preservation in soil structure, and thus, their removal becomes more difficult [9, 10].

Bioavailability of PAHs in the soil is one of the key factors that restrict the progress of biodegradation processes [11]. To increase the bioavailability of hydrophobic organic contaminants, surfactants can be used, which may influence the changes in the properties of bacterial cell surface or increase the dissolution and emulsification of hydrocarbons as well as release the substances bound in the porous substrate in the contaminated soil [12]. One of the recent advancements in this field is the use of natural surfactants, the so-called biosurfactants, including rhamnolipids, the hydrophilic part of which is composed of rhamnose molecules, whereas the hydrophobic part is composed of β-hydroxydecanoic acid [13]. *Pseudomonas* species, in particular, *Pseudomonas aeruginosa*, are the major producer of rhamnolipids. These compounds have also been detected in the cultures of *Acinetobacter*, *Pseudoxanthomonas*, *Enterobacter*, and *Pantoea* [14].

One of the highly sensitive indicators of the effect of petroleum derivatives on soil is its microbial activity [15]. The use of microbial processes in the analysis of soil environment allows to assess its ecological status [16]. Apart from that, biological parameters such as respiratory activity, live organism biomass content, and enzymatic activity are more sensitive and better explain the status of the soil environment than physicochemical properties [17].

Therefore, the objective of the present study was microbiological and biochemical assessment of the possibility of using rhamnolipids to control the effect of coal tar creosote on soil environment.

## 2 Materials and methods

### 2.1 Chemical reagents

Both coal tar creosote types (type C and type GX-Plus) were obtained from a single railroad tie treatment plant in Poland. Their basic properties are presented in Table 1. Rhamnolipids, 95%, produced by *Pseudomonas aeruginosa*, were purchased from Sigma-Aldrich. Following the instructions of the manufacturer, this product was enhanced by hydrocarbon degradation. The remaining reagents used for analyses were also obtained from Sigma-Aldrich. To prepare the solutions, deionized water (HLP Smart 2000 demineralizer, Hydrolab) with a mean conductivity of 0.15 μS·cm^−1^ and surface tension of 72.3 mN·m^−1^ at 25 °C was used.

**Table 1:** Comparison of properties of coal tar creosote types

Properties	Type C	GX-Plus
Density in 20°C [g·cm^−3^]	1.12	1.02
Water content [%]	0.22	0.81
Content of phenols extracted with water [%]	1.09	1.11
Content of benzo(a)pyrene [mg·kg^−1^]	12.08	5.78

### 2.2 Experiment design

The experiment was conducted on soil samples collected from the arable-humus horizon (0–20 cm) of chernozems in the Pyrzyce Plain (53°15’N, 14°92’E) under laboratory conditions. According to the classification of the United States Department of Agriculture, it was soil with a granulometric composition of sandy clay loam. The content of particular a fraction of the soil was as follows: sand (0.05–2 mm), 531.2 g·kg^−1^; silt (0.002–0.05 mm), 189.7 g·kg^−1^; and clay (<0.002 mm), 279.1 g·kg^−1^. The soil contained C_org_ of 33.81 g·kg^−1^ and N_tot_ of 2.74 g·kg^−1^, and the pH value of soil in 1 M KCl solution was 7.13. The collected soil was air-dried, sieved through a 2-mm mesh sieve, and divided into 1 kg samples.

The experiment was conducted in triplicate. The experimental factors were as follows: (1) coal tar creosote type (C type and GX-Plus type); (2) dose of coal tar creosote (0 and 10 g·kg^−1^), (3) dose of rhamnolipids (0, 10, 100, and 1000 mg·kg^−1^), and (4) incubation time of 1, 7, 21, and 63 days. Soil sample humidity was adjusted to 60% maximum water holding capacity, and the samples were incubated in tightly closed glass containers at 20°C.

During the experiment, the microbial biomass content and the activity of enzymes in the soil samples were measured in all the three subsequent replications.

### 2.3 Determination of microbial biomass content

The substrate-induced respiration method (SIR) was used to determine the microbial biomass content. Soil samples weighing 10 g were homogenized with glucose and talc mixture in 1:5 ratio. Soil samples were transferred to Ultragas U4S analyzer to measure the level of carbon dioxide emission over 3 h. After a particular time, the released CO_2_ was measured in mm. The obtained values were recalculated as cm^3^ CO_2_ released in an hour, and the amount of microbial biomass in soil was calculated based on the formula given by Anderson and Domsch [18].

### 2.4 Determination of soil enzyme activities

The activity of dehydrogenases (DHA) [EC 1.1.1] in soil samples was determined according to the method of Casida et al. [19]. This method includes incubation of soil in buffered (pH 7.6) 2,3,5-triphenyltetrazolium chloride (TTC), which is reduced by DHA to colored, water-insoluble triphenylformazan (TPF). By replacing oxygen and other naturally occurring acceptors, TTC accepts electrons and protons released by DHA from the oxidized organic compounds. After incubation, TPF was extracted from soil using ethanol and spectrophotometrically confirmed at the wavelength of λ = 485 nm.

The activity of alkaline phosphatase (ALP) [EC 3.1.3.1] and acid phosphatase (ACP) [EC 3.1.3.2] was determined using the method of Tabatabai and Bremner [20], with buffered (pH 11 for ALP; pH 6.5 for ACP) *p*-nitrophenyl phosphate solution. *p*-Nitrophenol (*p-*NP) obtained by the catalytic reaction with a phosphatase enzyme was extracted, stained with sodium hydroxide, and spectrophotometrically determined at the wavelength of λ = 400 nm.

Urease (URE) [EC 3.5.1.5] activity was determined using the method of Kandeler and Gerber [21]. This method includes incubation of soil with a buffered (pH 10) urea solution, which is then decomposed to ammonia and carbon dioxide. The released ammonia was collected using potassium chloride solution, and its content was determined by a modified Berthelot reaction. The determination process involves the reaction of ammonia with sodium salicylate in the presence of sodium dichloroisocyanurate (with sodium nitroprusside as the catalyst), which forms a green-stained complex under alkaline conditions, confirmed spectrophotometrically at a wavelength of λ = 690 nm

The UV-Vis 1800 spectrophotometer (Shimadzu, Kyoto, Japan) was used for enzymatic activity analysis. The obtained results were calculated from calibration curves, the standards for which were TPF, *p-*nitrophenol (*p-*NP), and ammonium chloride for dehydrogenases, phosphatases, and URE, respectively.

### 2.5 Data analysis

The obtained results were calculated with the formulas provided by Kaczyńska et al. [22] and were presented as coal tar creosote impact factor (*IFC*) and rhamnolipids impact factor (*IFR*) on the determined parameters:

$$IFC=\frac{A_{C}}{A_{0}}$$

$$IFR=\frac{A_{R}}{A}$$

in which: *IFC* is the coal tar creosote impact factor, *IFR* is the rhamnolipids impact factor, *A_C_* is the value of the determined parameter of soil contaminated with coal tar creosote, *A*_0_ is the value of the determined parameter of uncontaminated soil, *A_R_* is the value of the determined parameter of soil subjected to the effect of rhamnolipids, and *A* is the value of the determined parameter of soil not contaminated or contaminated with coal tar creosote.

The calculated *IFC* and *IFR* values were subjected to two-way analysis of variance test. For *IFC*, the variable factors were coal tar creosote type and incubation time, whereas for *IFR*, the variable factors were type and dose of coal tar creosote. In the latter case, the variance analysis was performed independent of each measurement date. Subsequently, the mean values were compared using a posthoc Tukey’s HSD (honest significant difference) test with significance at *p* = 0.05.

To determine which of the variable factors has the greatest effect on coal tar creosote and rhamnolipid impact factors, a η^2^ analysis was performed to determine the ratio of variance of a dependent variable to an independent variable – predictor [23]. The analyses were performed independently for each coal tar creosote type.

Moreover, Pearson’s linear correlation coefficients were calculated between the impact factor values of coal tar creosote and rhamnolipids for the determined parameters by using the Bonferroni correction [24].

The final *IFC* and *IFR* values were interpreted using principal component analysis (PCA). PCA was used because of the existence of four independent factors, and their interactions could have stemmed from the existence of one or more common factors. PCA was used to reduce the number of variables influencing the determined microbiological and biochemical parameters as well as to reveal the regularities between these variables. It consists in determining variables constituting a linear combination of the tested variables [25]. The application of PCA enabled a precise determination of the scale of changes of coal tar creosote and rhamnolipids impact factors on the soil environment [26].To perform statistical analyses, Statistica 13.3 software (Statsoft Inc.) was used.

## 3 Results

### 3.1 Effect of coal tar creosote on microbial biomass content and enzymatic activity in soil

The average content of microbial biomass and activity of enzymes in soil not subjected to the effect of coal tar creosote and rhamnolipids are presented in Table 2.

**Table 2:** Mean microbial biomass content and anzymatic activity in soil non-treated with coal tar creosote and rhamnolipids

Parameter	Unit	Activity
Biomass	mg C·kg^-1^ DM	12.728 ± 0.661
DHA	mg TPF·kg^-1^ DM·h^-1^	3.438 ± 0.232
ACP	mg *p*-NP·kg^-1^ DM·h^-1^	122.913 ± 5.489
ALP	mg *p*-NP·kg^-1^ DM·h^-1^	254.414 ± 14.819
URE	mg N-NH_4_·kg^-1^ DM·h^-1^	46.207 ± 2.360

DHA dehydrogenases, ACP acid phosphatase, ALP alkaline phosphatase, URE urease; TPF triphenylformazan, *p*-NP *p*-nitrophenol

**Figure 1:**
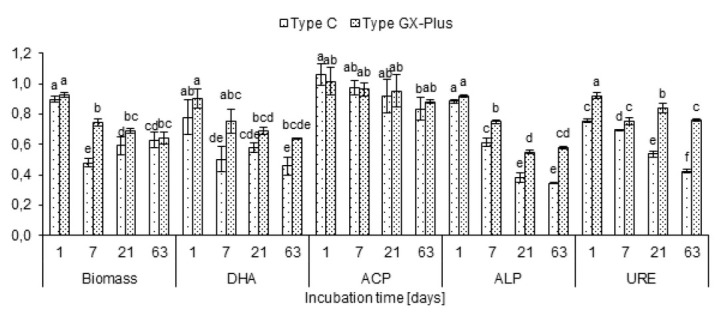
Indices of coal tar creosote effect (*IFC*) on microbial biomass and enzymatic activity in soil; DHA dehydrogenases, ACP acid phosphatase, ALP alkaline phosphatase, URE urease; the same letters for each parameter are assigned to the same homogenous group (post-hoc Tukey HSD test) at the significance level *p*=0.05

Contamination with coal tar creosote primarily resulted in reduced content of microbial biomass and enzymatic activity of soil. On the day 1, the *IFC* values were typically close to 1. Only for URE, the soil contamination with coal tar creosote type C resulted in a clear decrease in the activity of this enzyme (*IFC* = 0.75). On the subsequent measurement dates for all determined parameters, with the exception for ACP, a continuous decrease in *IFC* was observed. The strongest negative effect of coal tar creosote was found for microbial biomass content, ALP, and URE. Furthermore, in the majority of cases, coal tar creosote type C showed significantly higher inhibition of determined parameters than coal tar creosote type GX-Plus (Figure 1).

### 3.2 Effect of rhamnolipids on microbial biomass content in uncontaminated soil and soil contaminated with coal tar creosote

After the addition of rhamnolipids to uncontaminated soil, the *IFR* values were typically close to 1, which indicates a minor effect of rhamnolipids on microbial biomass content. In addition, no significant differences were found between *IFR* values depending on the dose of rhamnolipids. However, in soil contaminated with coal tar creosote type C, the addition of rhamnolipids at a dose of 100 mg·kg^−1^ DM resulted in a significant increase in microbial biomass content as compared to uncontaminated soil on all measurement dates. However, following the addition of rhamnolipids at the dose of 1000 mg·kg^−1^ DM, a significant increase in microbial biomass content occurred only on day 7. In soil contaminated with coal tar creosote type GX-Plus, a statistically significant increase in the microbial biomass content as compared to uncontaminated soil was observed only after the addition of rhamnolipids at doses of 100 and 1000 mg·kg^−1^ DM only on days 21 and 63. Furthermore, the observed increase in the content did not differ significantly between different rhamnolipids doses (Table 3).

**Table 3:** Indices of rhamnolipids effects (*IFR*) on microbial biomass content in uncontaminated soil and soil contaminated with coal tar creosote

Doses of rhamnolipids	Incubation time [days]			
	1	7	21	63
Uncontaminated soil				

10 mg·kg^−1^	1.008 ± 0.093^ab^	1.044 ± 0.099^bc^	0.947 ± 0.049^cd^	0.966 ± 0.015^cd^
100 mg·kg^−1^	0.961 ± 0.092^b^	1.012 ± 0.101^c^	0.872 ± 0.067^d^	0.935 ± 0.031^d^
1000 mg·kg^−1^	1.068 ± 0.028^ab^	1.032 ± 0.099^c^	1.048 ± 0.029^bcd^	1.052 ± 0.031^cd^

Soil contaminated with coal tar creosote type C				

10 mg·kg^−1^	1.050 ± 0.057^ab^	1.012 ± 0.041^c^	1.091 ± 0.103^bc^	1.038 ± 0.097^cd^
100 mg·kg^−1^	1.181 ± 0.110^a^	1.256 ± 0.101^ab^	1.239 ± 0.115^ab^	1.423 ± 0.118^a^
1000 mg·kg^−1^	1.026 ± 0.017^ab^	1.417 ± 0.016^a^	1.145 ± 0.066^abc^	1.167 ± 0.016^bc^

Soil contaminated with coal tar creosote type GX-Plus				

10 mg·kg^−1^	1.001 ± 0.044^ab^	1.014 ± 0.014^c^	0.982 ± 0.021^cd^	0.982 ± 0.024^cd^
100 mg·kg^−1^	1.046 ± 0.056^ab^	1.191 ± 0.049^bc^	1.222 ± 0.025^ab^	1.282 ± 0.042^ab^
1000 mg·kg^−1^	1.030 ± 0.028^ab^	1.215 ± 0.028^abc^	1.323 ± 0.024^a^	1.411 ± 0.028^a^

the same letters for each day are assigned to the same homogenous group (post-hoc Tukey HSD test) at the significance level *p*=0.05

### 3.3 Effect of rhamnolipids on DHA activity in uncontaminated soil and soil contaminated with coal tar creosote

The impact factor of rhamnolipids on DHA activity in uncontaminated soil was close to 1, indicating a minor effect of rhamnolipids on DHA activity. Only on day 1 after rhamnolipids addition at a dose of 1000 mg·kg^−1^ DM, a slightly higher *IFR* value was found, but it did not differ significantly from the values obtained after the addition of lower rhamnolipid doses. However, in soil contaminated with coal tar creosote type C, the *IFR* values after the addition of rhamnolipids were significantly higher than that in uncontaminated soil only on day 7 for the dose of 100 mg·kg^−1^ DM and on days 7 and 63 for the dose of 1000 mg·kg^−1^ DM. In soil contaminated with coal tar creosote type GX-Plus, the *IFR* values after the addition of rhamnolipids were significantly higher than that in uncontaminated soil only for dose 1000 mg·kg^−1^ DM on day 63 (Table 4).

**Table 4:** Indices of rhamnolipids effects (*IFR*) on activity of dehydrogenases in uncontaminated soil and soil contaminated with coal tar creosote

Doses of rhamnolipids	Incubation time [days]			
	1	7	21	63
Uncontaminated soil				

10 mg·kg^−1^	1.027 ± 0.100^a^	0.992 ± 0.052^c^	1.029 ± 0.031^a^	0.999 ± 0.039^c^
100 mg·kg^−1^	1.034 ± 0.019^a^	1.013 ± 0.094^c^	1.045 ± 0.096^a^	1.029 ± 0.088^bc^
1000 mg·kg^−1^	1.064 ± 0.084^a^	1.002 ± 0.035^c^	1.039 ± 0.099^a^	1.026 ± 0.073^bc^

Soil contaminated with coal tar creosote type C				

10 mg·kg^−1^	1.063 ± 0.078^a^	1.020 ± 0.093^c^	1.037 ± 0.076^a^	1.261 ± 0.115^abc^
100 mg·kg^−1^	1.092 ± 0.103^a^	1.271 ± 0.115^ab^	1.170 ± 0.087^ab^	1.379 ± 0.108^ab^
1000 mg·kg^−1^	1.072 ± 0.076^a^	1.427 ± 0.121^a^	1.176 ± 0.115^ab^	1.431 ± 0.126^a^

Soil contaminated with coal tar creosote type GX-Plus				

10 mg·kg^−1^	1.005 ± 0.034^a^	0.997 ± 0.033^c^	1.020 ± 0.049^a^	0.952 ± 0.024^c^
100 mg·kg^−1^	1.017 ± 0.085^a^	1.151 ± 0.040^bc^	1.218 ± 0.113^ab^	1.280 ± 0.017^abc^
1000 mg·kg^−1^	1.022 ± 0.076^a^	1.196 ± 0.066^abc^	1.325 ± 0.074^a^	1.398 ± 0.099^a^

the same letters for each day are assigned to the same homogenous group (post-hoc Tukey HSD test) at the significance level *p=*0.05

### 3.4 Effect of rhamnolipids on phosphatase activities in uncontaminated soil and soil contaminated with coal tar creosote

In the uncontaminated soil after the addition of rhamnolipids at doses 10 and 100 mg·kg^−1^ DM, the *IFR* values for acid and alkaline phosphatase were close to 1.

The addition of rhamnolipids at a dose of 1000 mg·kg^−1^ DM typically resulted in increased *IFR* value. However, these values did not differ significantly from those obtained for lower biosurfactant doses.

For ACP in soil contaminated with coal tar creosote type C compared with uncontaminated soil, a significant increase in the *IFR* value was recorded only for the dose of 1000 mg·kg^−1^ DM on days 1, 7, and 21, whereas in soil contaminated with coal tar creosote type GX-Plus, the *IFR* values were significantly higher than those obtained for uncontaminated soil after the addition of rhamnolipids at a dose of both 100 and 1000 mg·kg^−1^ DM on days 7, 21, and 63 (Table 5).

**Table 5:** Indices of rhamnolipids effects (*IFR*) on activity of acid phosphatase in uncontaminated soil and soil contaminated with coal tar creosote

Doses of rhamnolipids	Incubation time [days]			
	1	7	21	63
Uncontaminated soil				
10 mg·kg^−1^	1.019 ± 0.083^bc^	1.001 ± 0.008^e^	1.007 ± 0.007^d^	0.999 ± 0.042^d^
100 mg·kg^−1^	1.020 ± 0.068^bc^	1.067 ± 0.037^de^	1.014 ± 0.023^d^	1.067 ± 0.015^cd^
1000 mg·kg^−1^	1.112 ± 0.067^b^	1.050 ± 0.030^de^	1.102 ± 0.097^cd^	1.161 ± 0.056^bcd^

Soil contaminated with coal tar creosote type C				
10 mg·kg^−1^	0.910 ± 0.036^c^	1.093 ± 0.045^de^	1.113 ± 0.031^cd^	1.104 ± 0.035^cd^
100 mg·kg^−1^	1.063 ± 0.045^bc^	1.133 ± 0.032^cd^	1.147 ± 0.060^bcd^	1.193 ± 0.032^bc^
1000 mg·kg^−1^	1.293 ± 0.015^a^	1.428 ± 0.044^a^	1.513 ± 0.104^a^	1.206 ± 0.104^bc^

Soil contaminated with coal tar creosote type GX-Plus				
10 mg·kg^−1^	1.103 ± 0.047^b^	1.003 ± 0.063^e^	1.087 ± 0.059^cd^	1.133 ± 0.070^bcd^
100 mg·kg^−1^	1.010 ± 0.053^bc^	1.243 ± 0.024^bc^	1.213 ± 0.044^bc^	1.277 ± 0.066^ab^
1000 mg·kg^−1^	1.100 ± 0.046^b^	1.273 ± 0.080^b^	1.317 ± 0.032^b^	1.377 ± 0.045^a^

the same letters for each day are assigned to the same homogenous group (post-hoc Tukey HSD test) at the significance level *p*=0.05

The changes in *IFR* values were slightly different for ALP. In soil contaminated with coal tar creosote type C, a significant increase in the impact factor was observed after the addition of rhamnolipids at doses of 100 and 1000 mg·kg^−1^ DM on days 7 and 21, as compared to uncontaminated soil. Similar changes were observed in *IFR* values for all doses in soil contaminated with coal tar creosote type GX-Plus. However, the stimulating effect of the dose of 1000 mg·kg^−1^ DM was retained up to day 63 (Table 6).

**Table 6:** Indices of rhamnolipids effects (*IFR*) on activity of alkaline phosphatase in uncontaminated soil and soil contaminated with coal tar creosote

Doses of rhamnolipids	Incubation time [days]			
	1	7	21	63
Uncontaminated soil				
10 mg·kg^−1^	1.002 ± 0.093^bc^	0.983 ± 0.054^c^	1.025 ± 0.072^d^	1.030 ± 0.090^cd^
100 mg·kg^−1^	0.998 ± 0.069^bc^	0.968 ± 0.050^c^	1.004 ± 0.033^d^	1.018 ± 0.087^cd^
1000 mg·kg^−1^	1.059 ± 0.099^abc^	1.063 ± 0.021^c^	1.100 ± 0.023^cd^	1.142 ± 0.082^bcd^

Soil contaminated with coal tar creosote type C				
10 mg·kg^−1^	1.001 ± 0.017^bc^	1.070 ± 0.010^c^	1.013 ± 0.021^d^	0.993 ± 0.047^d^
100 mg·kg^−1^	1.130 ± 0.013^ab^	1.231 ± 0.024^b^	1.183 ± 0.009^c^	1.250 ± 0.042^abc^
1000 mg·kg^−1^	1.127 ± 0.057^ab^	1.293 ± 0.093^ab^	1.407 ± 0.006^b^	1.281 ± 0.018^ab^

Soil contaminated with coal tar creosote type GX-Plus				
10 mg·kg^−1^	0.917 ± 0.025^c^	1.012 ± 0.051^c^	1.093 ± 0.015^cd^	1.061 ± 0.036^bcd^
100 mg·kg^−1^	1.140 ± 0.052^ab^	1.380 ± 0.046^a^	1.351 ± 0.066^b^	1.238 ± 0.092^abc^
1000 mg·kg^−1^	1.241 ± 0.039^a^	1.362 ± 0.026^ab^	1.531 ± 0.055^a^	1.436 ± 0.117^a^

the same letters for each day are assigned to the same homogenous group (post-hoc Tukey HSD test) at the significance level *p*=0.05

### 3.5 Effect of rhamnolipids on URE activity in uncontaminated soil and soil contaminated with coal tar creosote

Similarly, the *IFR* values for the microbial biomass content and activity of other enzymes after rhamnolipids addition to uncontaminated soil at a dose of 10 mg·kg^−1^ DM were close to 1. However, in contrast to the above discussed microbiological and biochemical parameters of uncontaminated soil, significantly higher *IFR* values for URE were recorded after the addition of rhamnolipids at doses of 100 and 1000 mg·kg^−1^ DM than those at lower doses of these biosurfactants. By comparing the *IFR* values for soil contaminated with coal tar creosote with that for uncontaminated soil, a significant increase in the URE activity was found on day 7 after the application of rhamnolipids at doses of 10 and 1000 mg·kg^−1^ DM and on days 21 and 63 after the addition of these biosurfactants at doses of 100 and 1000 mg·kg^−1^ DM. However, in soil contaminated with coal tar creosote type GX-Plus, a significant stimulating effect of rhamnolipids on URE activity was found for the dose of 10 mg·kg^−1^ DM on days 7 and 63, at the dose of 100 mg·kg^−1^ DM on days 21 and 63, and at the dose of 1000 mg·kg^−1^ DM on days 7, 21, and 63. Finally, on the last measurement date, the calculated *IFR* values for the dose of 1000 mg·kg^−1^ DM were significantly higher than those for the dose of 10 mg·kg^−1^ DM (Table 7).

**Table 7:** Indices of rhamnolipids effects (*IFR*) on activity of urease in uncontaminated soil and soil contaminated with coal tar creosote

Doses of rhamnolipids	Incubation time [days]			
	1	7	21	63
Uncontaminated soil				
10 mg·kg^−1^	0.903 ± 0.089^c^	0.904 ± 0.071^d^	0.991 ± 0.078^cd^	1.028 ± 0.072^ef^
100 mg·kg^−1^	0.990 ± 0.058^bc^	1.134 ± 0.093^bc^	1.066 ± 0.058^cd^	0.994 ± 0.028^f^
1000 mg·kg^−1^	1.077 ± 0.095^abc^	1.157 ± 0.066^bc^	1.104 ± 0.082^c^	1.040 ± 0.071^def^

Soil contaminated with coal tar creosote type C				
10 mg·kg^−1^	0.968 ± 0.073^bc^	1.088 ± 0.033^c^	0.934 ± 0.051^d^	1.133 ± 0.012^cde^
100 mg·kg^−1^	1.139 ± 0.101^ab^	1.220 ± 0.027^bc^	1.291 ± 0.018^b^	1.243 ± 0.035^bc^
1000 mg·kg^−1^	1.078 ± 0.029^b^	1.498 ± 0.028^a^	1.344 ± 0.022^b^	1.374 ± 0.034^a^

Soil contaminated with coal tar creosote type GX-Plus				
10 mg·kg^−1^	0.978 ± 0.054^bc^	1.092 ± 0.059^c^	1.104 ± 0.024^c^	1.151 ± 0.041^cd^
100 mg·kg^−1^	1.116 ± 0.038^ab^	1.252 ± 0.049^b^	1.388 ± 0.017^ab^	1.228 ± 0.031^bc^
1000 mg·kg^−1^	1.221 ± 0.009^a^	1.416 ± 0.071^a^	1.504 ± 0.006^a^	1.323 ± 0.028^ab^

the same letters for each day are assigned to the same homogenous group (post-hoc Tukey HSD test) at the significance level *p*=0.05

### 3.6 Assessment of the level of experimental factor impact on the determined microbiological and biochemical parameters of soil

The η^2^ analysis was performed for each coal tar creosote type. For both coal tar creosote types, the strongest effect of rhamnolipid dose on the formation of all the determined biological parameters was found. Moreover, a strong effect was also found for the interaction between the coal tar creosote dose and rhamnolipids dose. For both coal tar creosote types, the changes in ACP activity were also considerably induced by the contaminant dose (Tables 8 and 9). However, a strong effect of the dose of coal tar creosote type GX-Plus was also found for URE activity.

**Table 8:** Percentage share of observed variability factors η^2^ for coal tar creosote type C

Variable factor	Biomass	DHA	ACP	ALP	URE
CD	3.012	1.157	27.471	0.262	0.746
RD	44.789	48.783	46.793	53.094	55.628
T	0.774	1.412	1.459	0.158	2.804
CD × RD	44.419	40.781	15.380	39.402	35.441
CD × T	0.553	2.401	3.186	1.447	0.747
RD × T	2.352	2.105	2.222	3.003	2.388
CD × RD × T	3.159	2.615	2.995	2.388	2.037
Error	0.943	0.746	0.494	0.246	0.209

CD coal tar creosote dose, RD rhamnolipid dose, T day of incubation, DHA dehydrogenases, ACP acid phosphatase, ALP alkaline phosphatase, URE urease

**Table 9:** Percentage share of observed variability factors η^2^ for coal tar creosote type GX-Plus

Variable factor	Biomass	DHA	ACP	ALP	URE
CD	7.312	0.973	35.099	9.301	24.199
RD	42.841	50.075	41.141	49.803	45.015
T	0.672	1.270	3.639	0.674	3.855
CD × RD	39.913	37.786	11.203	34.805	22.487
CD × T	2.013	1.680	2.385	0.876	3.855
RD × T	3.361	3.551	3.566	2.363	1.156
CD × RD × T	3.519	3.891	2.335	1.869	0.980
Error	0.369	0.775	0.632	0.310	0.248

CD coal tar creosote dose, RD rhamnolipid dose, T day of incubation, DHA dehydrogenases, ACP acid phosphatase, ALP alkaline phosphatase, URE urease

### 3.7 Relationships between microbiological and biochemical parameters

The calculated Pearson’s linear correlation coefficients demonstrated that a positive stimulation was observed between the changes caused by soil contamination with coal tar creosote and rhamnolipid addition (Table 10). The highest value of correlation coefficient was determined between ALP and URE activity changes (*r* = 0.796).

**Table 10:** Współczynniki korelacji liniowej Pearsona pomiędzy zmianami microbial biomass content i enzymatic activity w glebie po dodaniu coal tar creosote i ramnolipidów

Parameter	Biomass	DHA	ACP	ALP	URE
Biomass	-	0.626*	0.594*	0.697*	0.664*
DHA		-	0.551*	0.601*	0.640*
ACP			-	0.718*	0.703*
ALP				-	0.796*
URE					-

DHA – dehydrogenases, ACP – acid phosphatase, ALP – alkaline phosphatase, URE – urease; *denotes significant coefficients at the Bonferroni-corrected level (*p*≤0.00208)

**Figure 2:**
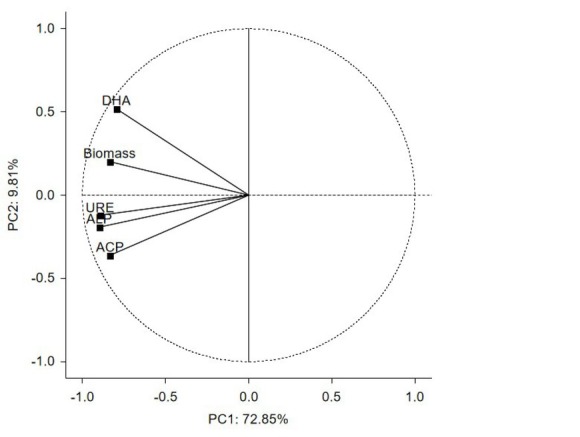
Microbial biomass content and activity of enzymes in soil treated with coal tar creosote and rhamnolipids as presented by the PCA method: DHA – dehydrogenases, ACP – acid phosphatase, ALP – alkaline phosphatase, URE - urease

The effect of coal tar creosote and rhamnolipids on microbial biomass content and enzymatic activity in soil was further explained by principal component analysis (PCA). PCA was used due to the existence of four interlinked factors, and their interactions could have stemmed from the existing one or more common factors. The distribution of vectors around the axis, which comprise of the first two factors, described 82.66% of total data variance: PC1 72.85%, PC2 9.81% (Figure 2). Changes in all determined parameters were negatively correlated with PC1. Consequently, DHA was also positively correlated with PC2 (Table 11).

**Table 11:** Pearson product-moment correlation coefficients between responses of changes in microbial biomass content and enzymatic activity in soil terated with coal tar creosote and rhamnolipids

Parameter	PC1	PC2
Biomass	-0.838	0.203
DHA	-0.794	0.518
ACP	-0.836	-0.360
ALP	-0.899	-0.190
URE	-0.896	-0.122

DHA dehydrogenases, ACP acid phosphatase, ALP alkaline phosphatase, URE urease;

## 4 Discussion

Contamination with petroleum derivatives, in particular, PAHs results in the need to search for various types of soil remediation. Our previous studies demonstrated the negative effect of coal tar creosote and volatile organic compounds emitted from wood treated with coal tar creosote on the activity of soil enzymes and the number of various microorganism groups [6, 27, 28]. The presented study results confirmed the degree of toxic effect of coal tar creosote on the microbiological and biochemical properties of soil. The study included the microbial biomass content and activity of the basic enzymes involved in carbon, phosphorus, and nitrogen cycle. Among the determined parameters, the strongest inhibiting effect of coal tar creosote was observed in microbial biomass content, ALP, and URE. Moreover, the toxic effect of coal tar creosote type C was higher than that of coal tar creosote type GX-Plus, which is in agreement with the results of a previous study [6]. This result stems from the fact that lower amounts of PAHs may be added to soil with coal tar creosote type GX-Plus than that with coal tar creosote type C [29].

Kaczyńska et al. [22] reported that DHA activity is a very good indicator of soil contamination. However, Bielińska et al. [30] demonstrated a negative correlation between the amounts of PAHs and activities of dehydrogenases and proteases, which indicates that enzymatic activity can be used as an indicator of the soil pollution with PAHs. In addition, numerous studies recorded the inhibitory effect of PAHs on the activity of various soil enzymes [6, 31, 32, 33, 34, 35]. The results we obtained further demonstrated the existence of a positive, significant correlation between the changes observed in microbial biomass content and enzymatic activity in soil treated with coal tar creosote and rhamnolipids.

The interest in biodegradation mechanisms and the presence of PAHs in the environment is associated with their low bioavailability and high durability in soil as well as their potential ecotoxicity. Considering the high hydrophobicity and solid-water distribution ratios, PAHs tend to interact with non-aqueous phases and organic matter in soil. As a consequence, they become unavailable to microbial decomposition [36]. Considering that the solubility of PAHs in water has an almost logarithmic decrease with the increase in molecular weight, PAHs with high molecular weight, the size of which ranges from five to seven rings, has become the object of special concern for the natural environment [37]. Posada-Baquero et al. [38] demonstrated that the application of rhamnolipids accelerated the decomposition of the absorbing PAHs’ fractions. Furthermore, it is interesting that BaP was subjected to a slow degradation after rhamnolipid addition.

The addition of rhamnolipids in uncontaminated soil typically had no influence on the microbiological and biochemical parameters. Liang et al. [39] reported that rhamnolipids may influence the secretion, activity, and even structure of enzymes. The use of these biosurfactants resulted in, for instance, stimulation of proteases and amylase during composting [40]. Despite the growing amount of information on the use of rhamnolipids in the bioremediation process of soils contaminated with petroleum derivatives, the mechanism of their action on microbial and biochemical processes has not yet been fully understood [38, 41].

In our study, no effect of rhamnolipids at the dose of 10 mg·kg^−1^ DM was observed, whereas the higher biosurfactant doses manifoldly stimulated the determined microbiological and biochemical parameters of soil contaminated with coal tar creosote. This may be due to the increasing bioavailability of hydrocarbons for the populations of microorganisms inhabiting soil under the influence of biosurfactants [42]. Furthermore, the η^2^ analysis conducted for both coal tar creosote types demonstrated that among all experimental factors, the dose of rhamnolipids had the strongest effect on the determined microbiological and biochemical parameters of soil. Compared with the earlier study on the changes of enzymatic activity of soil contaminated with coal tar creosote subject to stimulation with calcium peroxide [43] or low-molecular-weight organic acids [44], the use of rhamnolipids seems to be the most efficient bioremediation treatment option.

Kołwzan [12] reported that the information on the influence of biosurfactants on bioremediation process is frequently inconsistent. It was found that surfactants exhibit both positive and negative effects on bioremediation. The cause for such variability in results may be related to the repetition of identical experimental conditions. The course of bioremediation depends on a wide range of factors such as soil type, quality and quantity of microorganisms, type and amount of contaminants, or type and amount of surfactant. Therefore, it is difficult to foresee the effect of surfactant used in the bioremediation process. Practical application of the developed technologies is therefore only possible after performing preliminary experiments that enable the correct selection of the biosurfactant.

## 5 Conclusions

Contamination of soil with petroleum derivatives is currently one of the most important problems of ecotoxicology. Coal tar creosote poses a serious environmental threat because it contains high amounts of PAHs. Considerable amounts of PAHs can penetrate into the environment during the disposal of railroad ties treated with coal tar creosote. Thus, it is necessary to seek efficient bioremediation methods for soil contaminated with coal tar creosote as well as develop a reliable indicator of changes occurring in soil. The obtained results demonstrated that coal tar creosote type C exhibited higher toxicity toward microbial biomass content and enzymatic activity than type GX-Plus. The use of rhamnolipids, particularly at the dose of 1000 mg·kg^−1^ DM, stimulated the majority of determined microbiological and biochemical parameters. In addition, microbial biomass content and activity of alkaline phosphatase and URE were the best indicators of the effect of rhamnolipids in soil contaminated with coal tar creosote.
